# Solving the detour problem in navigation: a model of prefrontal and hippocampal interactions

**DOI:** 10.3389/fnhum.2015.00125

**Published:** 2015-03-20

**Authors:** Hugo J. Spiers, Sam J. Gilbert

**Affiliations:** ^1^Department of Experimental Psychology, UCL Institute of Behavioural Neuroscience, Division of Psychology and Language Sciences, University College LondonLondon, UK; ^2^UCL Institute of Cognitive Neuroscience, Division of Psychology and Language Sciences, University College LondonLondon, UK

**Keywords:** prediction error, hippocampus, planning, reinforcement learning, goals, virtual reality, place cells, artificial intelligence

## Abstract

Adapting behavior to accommodate changes in the environment is an important function of the nervous system. A universal problem for motile animals is the discovery that a learned route is blocked and a detour is required. Given the substantial neuroscience research on spatial navigation and decision-making it is surprising that so little is known about how the brain solves the detour problem. Here we review the limited number of relevant functional neuroimaging, single unit recording and lesion studies. We find that while the prefrontal cortex (PFC) consistently responds to detours, the hippocampus does not. Recent evidence suggests the hippocampus tracks information about the future path distance to the goal. Based on this evidence we postulate a conceptual model in which: Lateral PFC provides a prediction error signal about the change in the path, frontopolar and superior PFC support the re-formulation of the route plan as a novel subgoal and the hippocampus simulates the new path. More data will be required to validate this model and understand (1) how the system processes the different options; and (2) deals with situations where a new path becomes available (i.e., shortcuts).

## Introduction

### The Detour Problem and Background on Neural Systems for Navigation

Survival depends on being able to adapt behavior in response to changes in the world. One of the most common and problematic alterations to an environment is the discovery that the current path is blocked and a new path must be found. All motile animals must be able to adjust their movements to reach food and safety. Those animals with a sophisticated nervous system have evolved the capacity to learn a long-term internal representation of the environment. Such representations contain knowledge of the possible rewards associated with different path choices. While many studies have explored how neural systems support complex navigation or decision-making, surprisingly little is known about the brain regions that support adjusting a route when a forced detour is required.

Tolman (1948) provided some of the earliest behavioral studies where rats encountered a blocked path and were required find alternative routes. He found that rats with prior experience in the maze made impressively rapid adjustments in their path to the goal (Tolman and Honzik, 1930). From this, and other evidence, he argued that the rats had developed a flexible internal representation of the spatial relationships within the environment; a representation he termed a “cognitive map” (Tolman, 1948). Since this initial research a number of studies have documented the capacity of rodents and other animals to adapt their path to a goal in response to changes in the environment or barriers (Poucet et al., 1983; Chapuis, 1987; Chapuis et al., 1987; Jovalekic et al., 2011).

While the behavioral evidence for a cognitive map has been disputed (Bennett, 1996), the neural evidence has been compelling (O’Keefe and Nadel, 1978; Burgess, 2008; Spiers, 2012). Place cells, head-direction cells, grid cells and boundary cells (and their conjunctions) in hippocampal-parahippocampal regions provide evidence of an allocentric long-term spatial memory system (O’Keefe and Dostrovsky, 1971; Taube et al., 1990; Hafting et al., 2005; Sargolini et al., 2006; Lever et al., 2009). This system is capable of flexibly representing the geometric structure of the environment and maintaining it in long-term memory (Lever et al., 2002). Consistent with this, lesions to hippocampal and parahippocampal regions result in striking spatial deficits (e.g., Morris et al., 1982; Steffenach et al., 2005; Winocur et al., 2010).

Human neuroimaging and neuropsychological studies employing real or virtual reality (VR) environments have provided convergent evidence that hippocampal-parahippo-campal regions are engaged by navigation requiring an internal representation of the environment (Bohbot et al., 1998; Spiers et al., 2001a; Burgess, 2008; Spiers and Barry, 2015). This work has highlighted the importance of a network of brain regions for navigation that encompass not only hippocampal-parahippocampal structures but also retrosplenial cortex, posterior parietal cortex, cerebellum, prefrontal cortex (PFC) and striatum (see e.g., Spiers and Maguire, 2007a; Chadwick and Spiers, 2014; Ekstrom et al., 2014; Rondi-Reig et al., 2014; Spiers and Barry, 2015). These brain circuits appear to be central for navigation, but also to serve long-term memory for non-spatial information (see e.g., Moscovitch et al., 2005; Spiers, 2012). However, despite this substantial research with a wide variety of methods, few studies have explored one of the hallmarks of flexible navigation—the ability to take optimal detours when the shortest path is blocked.

## Theoretical Perspectives

### Model-Free and Model-Based Navigation Control Systems

In order for an animal to change its behavior in response to an obstructed path, it must have the capacity to: (a) detect the change to the environment; (b) inhibit the current action plan; and (c) select the next most appropriate course of action. In detecting the change it is also efficient for the animal to update its knowledge of the possible paths available for future journeys. The selection of the most appropriate course of action can rely on a range of information. If the goal happens to be visible along another path, a simple Pavlovian approach response would suffice to reach the goal (van der Meer et al., 2012). If, however, the goal is not visible but the environment or relationship to the goal is known, other mechanisms can be used to select the path. This may involve the generation of a new subgoal, which counter-intuitively, might require an initial movement *away* from the ultimate goal.

In formulating their theory of the hippocampus as a cognitive map, O’Keefe and Nadel (1978) argued that it was the capacity for flexible place-based (“locale”) navigation that distinguished the hippocampus from cue-guidance (“taxon”) based systems. Since this initial perspective a number of researchers have developed the idea of navigational guidance drawing on concepts in the reinforcement-learning framework (Sutton and Barto, 1998). A hippocampal navigation system that uses a representation of the paths in the environment has been conceptualized as providing “model-based” representation for a controller to guide navigation (Hasselmo, 2005; Lengyel and Dayan, 2007; Gustafson and Daw, 2011; Martinet et al., 2011; Simon and Daw, 2011; Hirel et al., 2013; Penny et al., 2013). This model-based system would allow for a “tree-search” of the possible paths to the goal. This would allow the animal to select the shortest most efficient path. This system contrasts with a cache-based habit (“model-free”) system capable of guiding navigation based on learned associations between actions and stimuli. This system is thought to involve the striatum (Gläscher et al., 2010; van der Meer and Redish, 2011) and is less computationally demanding because no tree-search or planning is required. Conversely model-free controllers of behavior suffer from being less flexible and more rigid in nature (Johnson et al., 2007; van der Meer et al., 2012). In addition it is possible that hippocampal regions may also determine the route by episodic retrieval of recent one-shot experiences, obviating the need for an elaborative tree-search and specifying the full route to the goal (Lengyel and Dayan, 2007).

In reinforcement learning theory (Sutton and Barto, 1998), learning systems make predictions about the future outcome of certain actions, such as taking one path over another. When these predictions deviate from the sensory information from the environment, such as when a path is blocked, the systems signal a prediction error that leads to updating in the representation of rewarded actions (Sutton and Barto, 1998). Prediction errors may arise due a variety of changes to the sensory stimuli, but not all of these will affect the current path of the animal to its goal. For example, a known bridge might be encountered which has changed its color, but the path across it remains the same, or the bridge may have be broken making the current planned path void. In the latter case the animal must find an alternative route, a detour, to the goal and update the reward associated with the action of taking a new path.

### A Homing Signal: Vector-Based Navigation

In contrast to reinforcement learning theory approaches to path selection, another source of information has been argued to guide navigation—a goal vector that combines the direction and Euclidean distance to the goal (Burgess and O’Keefe, 1996; Kubie and Fenton, 2009, 2012). In vector navigation the animal retrieves and monitors a vector between their location and the goal location (Kubie and Fenton, 2009, 2012). When returning to a recently vacated goal, a vector can be determined by computing self-motion information about distance traveled and rotations made. This is referred to as “path integration” or “dead reckoning” (see Etienne and Jeffery, 2004) and may (Worsley et al., 2001; Wolbers et al., 2007), or may not (Shrager et al., 2008; Kim et al., 2013), involve hippocampal-parahippocampal structures, perhaps depending on the distance navigated (Arnold et al., 2014). Such a homing system may also be used to guide navigation to locations retrieved from long-term memory (Kubie and Fenton, 2009, 2012). Recent fMRI evidence indicates that the entorhinal/subicular region encodes both the distance along vector to the goal (Spiers and Maguire, 2007b; Howard et al., 2014) and the direction to the goal relative to the environment’s axis (Chadwick et al., 2015). Such information about the direction to the goal would potentially be useful in determining the next most optimal route towards the goal (Kubie and Fenton, 2009, 2012). Neuroimaging data suggests an entorhinal Euclidean distance signal is dissociable from a hippocampal simulation of future paths (Howard et al., 2014; Spiers and Barry, 2015).

## Background on Prefrontal Cortex

### Caveats

A small number of human neuroimaging and rodent single unit studies have reported on how brain regions respond to forced detours. In the next section we discuss these, focusing on the PFC and the hippocampus. We note that some evidence suggests the posterior parietal cortex also plays an important role in flexibly responding to detours and route planning (Nitz, 2006, 2014; Spiers and Maguire, 2006; Rauchs et al., 2008; Whitlock et al., 2008; Calton and Taube, 2009; Viard et al., 2011; Howard et al., 2014) and spatial novelty detection (Howard et al., 2013). However, we do not discuss the role of the parietal cortex here because of the lack of single unit, lesion and neuropsychological evidence, and also because posterior parietal lobe responses have been less consistent in relevant neuroimaging studies (e.g., Maguire et al., 1998; Iaria et al., 2008). Future research will be important to address this limitation and provide sufficient data for a formal meta-analysis of neuroimaging studies.

### A Brief Primer on Prefrontal Function

The human frontal lobes have long been recognized to play an important role in adaptive, flexible behavior, and “executive functions” (e.g., Penfield and Evans, 1935; Luria, 1966; for reviews see Miller and Cohen, 2001; Gilbert and Burgess, 2008; Duncan, 2010; Shallice and Cooper, 2011; Passingham and Wise, 2012). For example, lesions to this region have been associated with impairments in inhibiting prepotent responses (Aron et al., 2003), switching flexibly from one behavior to another (Milner, 1963), goal-directed planning (Shallice, 1982), and strategy application (Shallice and Burgess, 1991). PFC is thought to support these abilities via modulatory interactions with posterior cortical regions and subcortical structures such as the basal ganglia and hippocampus (Miller and Cohen, 2001; Simons and Spiers, 2003; Preston and Eichenbaum, 2013).

One major research question has been whether the high-level control processes supported by the PFC can be fractionated, and if so to what extent these processes can be mapped onto distinct anatomical subdivisions. Evidence from functional neuroimaging has suggested that diverse cognitive demands can yield similar patterns of signal change in regions such as the dorsolateral PFC and anterior cingulate (Duncan and Owen, 2000; Duncan, 2010). However, other regions such as the anterior PFC appear to be recruited in a more circumscribed set of situations such as those involving multitasking (Roca et al., 2011). Furthermore, anterior PFC is itself a functionally heterogeneous region (Gilbert et al., 2006, 2010). Thus, we will consider below whether the processes involved in dealing with detours can be linked with specific regions of PFC, and to what extent these prefrontal regions may be considered to play related roles across spatial and non-spatial tasks.

## Neuroimaging Studies of Forced Detours during Navigation: A Prefrontal Affair

### Comparing Navigation Periods with and without Detours

**Figure 1:**
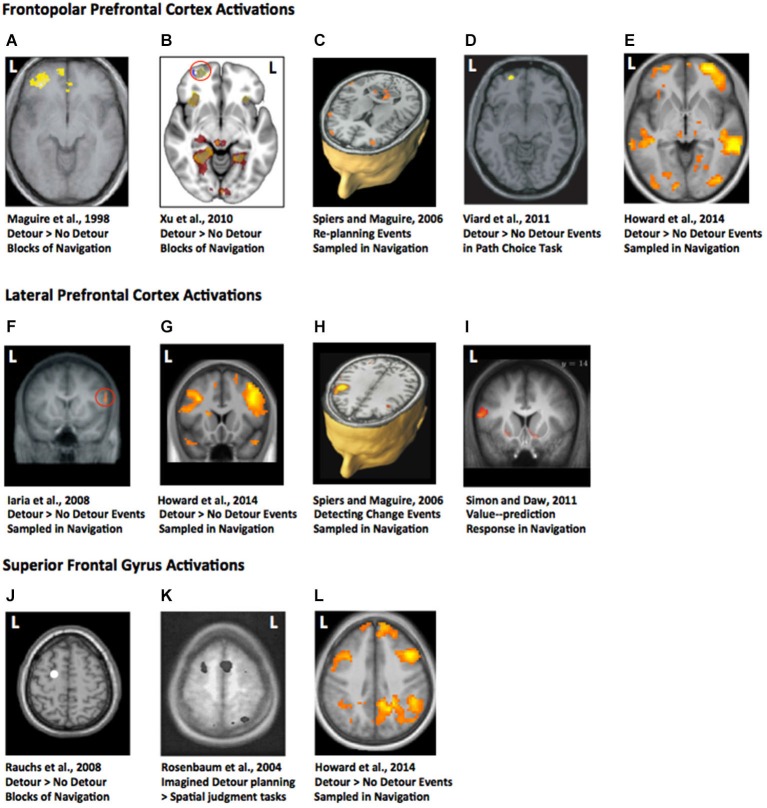
**Statistical parametric maps of brain imaging data in studies involving detours in navigation. (A–E)** frontopolar prefrontal cortex (PFC) activations. L = left. **(B)** (Xu et al., 2010): Areas in yellow indicate regions activated by detours more than non-detours. Areas in red indicate navigation more than line following. The red circle indicates a frontopolar region relatively selective to the detour condition. **(F–I)** Lateral prefrontal regions more active in a detour condition than a no-detour condition, or responsive to value prediction (Simon and Daw, 2011).** (J–L)** Superior frontal gyrus active more in a detour than control conditions. Images adapted from the articles cited under the images with permission. Image in **(J)** shows a coronal section of the canonical T1 image from SPM8 with a white marker indicating the location of the peak coordinate in the left frontal gyrus activation reported by Rauchs et al. (2008).

While numerous human neuroimaging studies have explored the brain regions involved in spatial navigation, only nine studies have examined the response to forced detours. These studies have all reported increased PFC activity when taking detours was compared to a control condition (see Table 1; Figure 1). This is consistent with the prediction that the PFC would be important for supporting flexible behavior in response to changing environmental contingencies and generating subgoals (e.g., Shallice and Burgess, 1991; Miller and Cohen, 2001; Spiers, 2008; Kim et al., 2011; Ullsperger et al., 2014). Based on theoretical considerations one might predict that the hippocampus would be more active when detours are required. This is because detours should be more demanding on memory retrieval and require some new learning. In contrast to this prediction, none of the nine studies that have explored detours found more hippocampal activity in response to detours. Moreover, two studies showed less activity in response to detours (Xu et al., 2010; Viard et al., 2011). We return to why this may be the case after discussing the pattern of responses in the PFC and the experimental design used in each study.

**Table 1:** **Prefrontal cortex activations in studies examining detours in spatial navigation tasks**.

Authors, year	Task	PFC Areas Active in Detour Condition
Maguire et al. (1998)	Navigate VR town learned 40–60 min prior to scanning. Analysis: Detour epochs > non-detour epochs.	L frontopolar PFC, L ventrolateral PFC
Rosenbaum et al. (2004, 2007)	Plan a route between two familiar real-world landmarks. Analysis: Comparison conditions, proximity judgments and route sequencing.	L superior frontal gyrus (BA6), R middle frontal gyrus (BA6)
Spiers and Maguire (2006)	Navigate in VR simulation of familiar city. Analysis: (1) Detection of changes in the environment, (2) Re-planning. Both were compared to baseline navigation periods.	(1) Detecting unexpected events: Bilateral lateral PFC, (2) Re-planning: Bilateral frontopolar PFC, R lateral PFC
Iaria et al. (2008)	Navigate VR paths with fences, which could change locations. Analysis: Detour events > non-detour events.	Right lateral PFC (BA 45 47/12)
Rauchs et al. (2008)	Navigate VR town learned 40–60 min prior to scanning. Analysis: Detour epochs > non-detour epochs.	L inferior frontal operculum Left superior frontal gyrus
Xu et al. (2010)	Navigate VR museum learned 40–60 min prior to scanning. Analysis: Detour epochs > non-detour epochs.	R middle frontal gyrus, L medial superior frontal gyrus
Viard et al. (2011)	Spatial decision making about which of two paths to take to reach a goal. Analysis: Detours events > non-detour events.	L frontopolar PFC (BA10), Bilateral medial PFC (BA6), R ventromedial PFC (BA9)
Simon and Daw (2011)	Navigate a grid-maze, with a changing one-way system, to reach rewarded locations. Behavior analyzed with a reinforcement learning model inversion to predict parameters associated with value coding.	Model-based planning representations of value: Lateral PFC
Howard et al. (2014)	Navigate a city region learned days prior to scanning. Analysis: (1) Detour events > non-detour events, (2) Detour events > Detours events in non-navigation control task. See Figure 3, for additional parametric analysis.	(1) Detour events > non-detour events: Bilateral frontopolar PFC, Bilateral lateral PFC. L superior frontal gyrus (2) Detour events > detour control events Bilateral frontopolar PFC, Bilateral lateral PFC. Bilateral superior frontal gyrus

*VR = Virtual reality. BA = Brodmann area. L = Left, R = Right. All used fMRI except Maguire et al. (1998), which used PET*.

The first neuroimaging study to explore the impact of forced detours recorded cerebral blood flow with positron emission tomography (PET) while subjects navigated a VR town learned just prior to scanning (Maguire et al., 1998). Three experimental tasks were examined with a block design. The tasks were: following arrows to a target destination (control condition), navigating the environment learned prior to testing (navigation condition), and navigating the environment taking detours caused by added barriers (detour condition). While the hippocampus was more active during the navigation condition compared to following arrows, it was not more active when the barriers were present. By contrast, prefrontal activity was not observed when navigation was compared to following arrows, but two regions of the PFC, frontal pole and middle frontal gyrus, were more active when subjects navigated the town with barriers than without barriers (Figure 1A; Table 1). This has been considered as evidence that while the hippocampus is a core region for navigation, its computations are not more needed when alternative routes need to be taken (Maguire et al., 1998). Rather the PFC regions are key in supporting the inhibition of prior planned routes and the forward planning of future decisions (Maguire et al., 1998; Spiers and Maguire, 2006, 2007a).

Since the study by Maguire et al. (1998) two other studies using a similar design with VR and fMRI have reported similar results (Rauchs et al., 2008; Xu et al., 2010). Both studies found PFC activity during the detours condition compared to the no-detours condition (Figure 1). Again, the hippocampus was not more active in the barriers condition compared to the no-barriers conditions. While Rauchs et al. (2008) reported a more widespread pattern of active brain regions, including superior frontal regions (Figure 1J), Xu et al. (2010) found a smaller subset more active in the barriers condition (Figure 1B). This difference may have related to differences in statistical thresholding and power, but both studies also reported some increased posterior parietal lobe (angular gyrus) activity in the detour condition.

### Event-Related Analysis of Detours: Consideration of the Prediction Error

A pitfall with the block designs, which compare activity during epochs with and without detours, is that the activity is not specific to the event of discovering a detour is needed. Instead it may relate to expectation prior to the detour and/or the experience in the period after taking the detour along a novel route. To determine how brain regions react to forced detours several studies have employed an event-related analysis. Spiers and Maguire (2006) recorded brain activity from London licensed taxi drivers as they navigated through a highly detailed virtual simulation of London (UK). Using a retrospective verbal report and a video replay it was possible to determine when the taxi drivers encountered unexpected changes to the environment (expectation violations) and events where they decided to re-plan their route. Re-planning and expectation violation events were not highly correlated since taxi drivers did not always report re-planning in the same moment they discovered the change to the environment (see Spiers and Maguire, 2008). Re-planning the route evoked more activity in frontopolar PFC than matched events when the taxi drivers reported simply traveling along (Figure 1C). Re-planning also elicited increased activity in a more posterior right lateral PFC region (Figure 1C). A similar right lateral region, extending more dorsally, was also active when subjects reported detecting unexpected changes to the environment (Figure 1H). This suggests it is possible that the frontopolar PFC regions may relate to re-planning paths during epochs involving forced detours, while a predominately right lateral region of PFC may also be specifically involved in the event of detecting the deviation from what was expected (Spiers and Maguire, 2006). Such a result is consistent with fMRI research using a range of paradigms that find lateral PFC regions are active when detecting occurrences of stimuli that deviate from expectations, particularly when those deviations have behavioral relevance (Fletcher et al., 2001; Corlett et al., 2004; Gläscher et al., 2010). This perspective is also supported by evidence that damage to lateral PFC disrupts the detection of novelty (e.g., Løvstad et al., 2012).

An important consideration when exploring the neural responses to forced detours is that not all changes in the environment require a detour. As noted earlier, if a familiar bridge is discovered to be painted a new color this would be surprising and generate sensory prediction errors, but it would not affect the prediction about what path will be accessible to the goal. To separate the neural responses to changes that require a new route from those that do not Iaria et al. (2008) scanned subjects whilst they navigated a virtual route round fences. On some journeys the fences changed position requiring a detour, on other journeys they changed to a new position that did not affect the current route to the goal. While detection of perceptual changes to the layout of the fences resulted in more posterior cortical responses (e.g., temporo-parietal junction), changes that required a change in the path to the goal resulted in right lateral PFC response (Figure 1F). This PFC region overlaps with the region showing increased activity to both re-planning (Figure 1C) and expectation violation (Figure 1H), providing further evidence that the lateral PFC is a region important for detecting prediction errors with behavioral relevance to the current route plan.

### Neural Responses when Detours are Expected

In the neuroimaging studies considered so far, the detours were surprising events, detected in periods of continuous navigation of the environment. Thus, it is possible that the posterior lateral PFC response may be in part due to the detection of change during on-going behavior. In support of this it is notable that in two studies no increased responses in lateral PFC were observed when subjects were expecting the detours on all, or half, of the trials and purely focused on the event of planning an alternate route (Rosenbaum et al., 2004, 2007; Viard et al., 2011). Rosenbaum et al. (2004, 2007) had subjects view photographs of pairs of landmarks in a familiar city (Toronto) and imagine navigating between them in the context that the main road linking the locations was blocked. Subjects also performed a range of other tasks, such as making proximity judgments about the landmarks with reference to a third landmark. Activity that distinguished the blocked-route tasks from the other spatial tasks was located in the superior PFC (Figure 1K), again consistent with the notion that PFC is particularly engaged by flexible behavior. However, unlike other studies considered in this review, the task investigated by Rosenbaum et al. (2004, 2007) did not involve inhibiting a previously established plan and developing a new plan, seeing as participants were merely instructed to find a route between two points having previously been told that an obvious route was not available. Thus, the superior PFC activation may have reflected a conflict effect between alternative routes under consideration, akin to conflict effects reported in non-spatial decision-making tasks that have reported activity in similar regions (BA6; Wendelken et al., 2009).

Further insights into prefrontal response to detours were provided by a task used by Viard et al. (2011) in which subjects made decisions on each trial about the path they would take to reach a man on the other side of a room. Half of the trials involved the need for a detour round a barrier, which was not pre-warned before the beginning of the trial. Thus, while detours were not surprising (occurring on 50% of trials), they could not be predicted on a trial-by-trial basis and therefore may have necessitated re-planning from the most direct route when they occurred. This may explain the frontopolar activity observed by Viard et al. (2011); Figure 1D, but not Rosenbaum et al. (2004, 2007).

### Model-Based Analysis of fMRI Data

As we discussed in the section on Model-free and model-based navigation control systems, when the goal to be navigated to cannot be seen, route choice may rely either on a retrieved map of the environment (a “model-based” representation) or on a learned association between the expected value and the action of choosing a particular path (a “model-free” representation)(van der Meer et al., 2012). In the studies discussed so far it was not possible to dissociate these possibilities. To address this Simon and Daw (2011) scanned subjects while they navigated a learned environment consisting of four rewarded goals randomly located in a virtual world composed of Manhattan-like 4 × 4 grid of 16 locations. Grid locations were connected by one-way doors. As subjects moved between locations each door had a 1/24 chance of reversing its direction. Thus, previously-available routes between locations might suddenly become impassible, requiring a detour along a new route. Simon and Daw (2011) fit the behavioral choices of the subjects to reinforcement learning models to estimate the changing representations of value associated with different choices made. For example, for a given participant, the choice to travel along a path from location one to two might be estimated to have a high value for the first 10 journeys because it was frequently chosen and provided a quick route to obtain reward. However, in the trials after one of the one-way doors on that route changed direction, the value of the choosing to travel along that path would diminish subsequently. In this scenario prediction-errors and value were correlated. One estimated set of values was determined assuming a model-free system; the other set was estimated assuming a model-based system. The authors found evidence that the subjects’ behavior was better captured by the model-based fit and that activity in a range of striatal regions tracked value as would be predicted from many prior fMRI studies on reinforcement learning (see O’Doherty et al., 2007). Additionally, lateral PFC regions extending into the frontal pole were correlated with the value of chosen paths derived from a model-based system (Figure 1I). This provides more precise evidence that lateral PFC regions represent the changing value of particular actions in response to alterations in the environment.

### Forced Detours in the Absence of Visual Cues

The method used by Simon and Daw (2011) provides a helpful approach to quantifying the impact of changes of the environment on behavior and internal representations. However, with the design used it was not simple to quantify the impact the detour had on the possible path to the goal, and like all the previous studies discussed, the change was correlated with a visual change in the structure of the environment (door entry signs changed). Furthermore, while Iaria et al. (2008) demonstrated that lateral PFC responses can be distinguished between perceptual changes that evoke a change in the route or not, they were still visually driven and the period after the detour was encountered differed from the non-detour events due to the change in path taken. In a recent fMRI study, Howard et al. (2014) examined: (a) the impact of forced detours on the representation of the distance to the goal; and (b) forced detours where no visual information signaled the need for a detour. Subjects learned, by studying maps and a two-hour walking tour, the layout of London’s (UK) Soho region. The next day subjects were scanned with fMRI while they watched a film-based first-person-view simulation of routes through the streets. During half of the routes the subject had to navigate to goal locations; for the other half the subjects just watched the routes and followed instructions about which path to select. In the navigation routes, just prior to each junction, subjects had to make a choice about the optimal path to the goal. The routes were fixed and subjects were told that most of the time the route would take the optimal path, but occasionally the route would take a forced detour. This meant that detours could only be distinguished from non-detours by the mismatch between choice prior to the turn and the outcome at the turn. Increased activity in frontopolar (Figure 1E), lateral (Figure 1G) and superior PFC (Figure 1L) regions reported in previous studies was found when detours were compared to non-detours. Similar regions were also found to be more active when detours in the navigation task were compared with equivalent events in the control routes (e.g., where a subject was instructed to select to turn left, but the route turned right). This supports the view that the PFC responses are not driven by visual changes in the environment but rather by the need to adapt the route plan. Notably, the study by Howard et al. had subjects navigate along paths only experienced once or twice before, and studied from maps, emphasizing the use of the model-based planning system. Thus, it seems likely that these responses are specific to the use of a model-based representation of the environment.

### Summary of Insights from Neuroimaging Studies

In summary, it is apparent that all neuroimaging studies exploring brain activity in response to forced detours reported increased PFC activity (Table 1). Frontopolar cortex appears to be region most consistently activated by the need to take detours compared to not having to take detours. More posterior lateral PFC regions are notably activated in response to detecting surprising events that necessitate detour planning. This response is consistent with the lateral PFC representing a model-based prediction error about the expectation that current planned route will succeed. Such a prediction-error signal could trigger inhibition of the ongoing navigation behavior (Chatham et al., 2012), leading to flexible route re-planning. Notably, we do not see reliable responses in the dorsomedial PFC/anterior cingulate or orbital frontal PFC regions. Other evidence indicates that these regions might be expected to play a role in aspects of navigation, such as coding the reward of reaching the goal (Feierstein et al., 2006; Spiers and Maguire, 2007b; Howard et al., 2014), “regret” in not obtaining reward (Steiner and Redish, 2014), dealing with overlapping routes (Brown et al., 2010; Brown and Stern, 2014) and strategy applications (Dahmani and Bohbot, 2015). Such processes may not be a key component in all detour taking and thus explain why such regions were less consistently responsive, or not responsive, to forced detours.

## How do Prefrontal Responses in Navigation Paradigms Relate to Responses in other Cognitive Tasks?

It is notable that a majority of studies investigating route re-planning in response to detours have reported prominent activation in frontopolar PFC (BA 10; Figures 1A–E). This is anterior to the lateral PFC regions most commonly activated by diverse cognitive demands (Duncan, 2013), suggesting that the processes involved in dealing with detours show some selectivity for this anterior region. Such a result would be consistent with neuroimaging investigations of non-spatial tasks, which have suggested that frontopolar regions are involved in “goal-subgoal integration” (Braver and Bongiolatti, 2002) or cognitive “branching” (Koechlin et al., 1999), i.e., the process of pursuing a subtask or subgoal whilst holding an overarching goal in mind (see also Ramnani and Owen, 2004). Conceptually, this fits well with a model whereby detours force participants to navigate towards a subgoal destination, whilst holding in mind an ultimate destination as an overall goal. Thus, both spatial navigation and non-spatial planning tasks implicate frontopolar PFC in subgoal processing. More broadly, dealing with forced detours would be a good example of a situation requiring attention to be balanced between internally-represented information (i.e., navigation goals and subgoals) and ongoing perceptual input as one moves through the environment. Frontopolar PFC has been proposed to play a key role in just such situations (Gilbert et al., 2005; Burgess et al., 2007).

A further point of consistency might be with Goel and Grafman’s (1995) neuropsychological study of the Tower of Hanoi planning task. Goel and Grafman argue that their frontal lobe patients’ difficulties with this task stem especially from the process of resolving a goal-subgoal conflict, particularly when pursuing a subgoal necessitates an initial move away from the ultimate goal state. Notably, of the 11 patients studied by Goel and Grafman for whom lesion localization in terms of Brodmann Areas (BA) was possible, nine had damage including (albeit not limited to) frontopolar region BA 10.

## Prefrontal Contributions from Single Unit and Lesion Studies

### Single Unit Recording and Lesion Studies in Rodents: A Short Tale

It would be highly beneficial if single unit recording or lesion studies in rodents and primates could help provide convergent evidence to support neuroimaging data. Alas, to our knowledge prefrontal responses to detours have not been characterized in rodents or non-human primates. The imperfect homology between primate and rodent PFC precludes making straightforward comparisons across species (Simons and Spiers, 2003). In rodents research on PFC contributions to navigation have focused on the prelimbic/infralimbic regions of the medial PFC, which receive mono-synaptic afferents from the hippocampus (Ferino et al., 1987). Neurons in medial PFC show activity related to the goal location (Hok et al., 2005) and medial PFC lesions impair switching from navigating from one learned goal to a new goal location (de Bruin et al., 1994; Granon and Poucet, 1995; Delatour and Gisquet-Verrier, 2000; Boulougouris et al., 2007) and switching between response and place strategies (Ragozzino et al., 1999a,b). Thus, it seems plausible that medial PFC damage would impair the inhibition of the current route and switching to a new route required in forced detour taking.

### Neuropscyhological Evidence from Humans: An Even Shorter Tale

In humans, anterior PFC lesions have been shown to result in impaired planning of routes to collect items from a set of different shops (Shallice and Burgess, 1991). Such deficits may relate more generally to poor application of strategy than to accommodating detours during navigation (Shallice and Burgess, 1991; Szczepanski and Knight, 2014). In relation to large-scale navigation, a lesion encompassing much of ventral medial PFC extending to frontopolar regions was found to result in an impaired ability to maintain the current spatial goal (Ciaramelli, 2008; Spiers, 2008). The patient often arrived at non-intended locations, showing a diminished ability to flexibly navigate across the town they were familiar with. Unfortunately there have been no reports of patients with frontal lobe damage being tested on their capacity to take optimal detours to a goal.

## A Role for the Hippocampus?

### Evidence from Neuropsychological Studies

Given the dominant theory that the hippocampus is essential for flexible navigation (O’Keefe and Nadel, 1978), one might expect it to play a prominent role in processing information to support detour taking. As noted above, in all nine neuroimaging studies reviewed none showed more activity when detours were required, compared to when they were not. Damage to the hippocampus in humans impairs the ability to learn and navigate a new environment (e.g., Spiers et al., 2001b), a problem noted in the case of the famous amnesic HM (Scoville and Milner, 1957). This makes testing patients on detour taking in novel environments not particularly informative. More relevant are tests of detour taking in environments pre-morbidly learned. Four studies have examined the ability of patients with hippocampal damage to navigate remotely learned environments and take detours (see for review Spiers and Maguire, 2007a). Patients EP (Teng and Squire, 1999), and KC (Rosenbaum et al., 2000), both with extensive medial temporal lobe damage, were tested on their ability to mentally describe routes between locations in their hometown with the requirement that they take detours to avoid a major road. Both patients, despite being densely amnesic, were able to perform this task as well as matched healthy controls, suggesting the hippocampus is not specifically needed for mentally planning routes in well-learned environments. However, more recently, different pattern of results was provided by a patient (HC) with congenital damage to their hippocampal anatomy (hippocampus, fornix and mammillary bodies). Patient HC was tested on her ability to provide route descriptions accommodating detours in a familiar environment (Rosenbaum et al., 2015). While she was able to describe routes that reached the destination, these routes were significantly longer than those described by the matched control subjects and her sketch maps suggested she had access to only schematic information about the environment. This suggests that gradual acquisition of the structure of the environment can occur in the context of congenital hippocampal damage, but that the hippocampus is needed to form detailed knowledge structures needed for accurate navigation and detour taking.

While the studies of patients HC, EP and KC have provided insights in the ability of hippocampal patients to perform mental navigation, they did not assess whether when the patient was able to actively navigate the environment. It is possible that cues in the environment might aid navigation, allowing patients with hippocampal damage to navigate remotely learned environments accurately. In order to test *in situ* navigation of a remotely learned environment Maguire et al. (2006) tested a retired London taxi driver with extensive bilateral hippocampal lesions (patient TT) on his ability to navigate the virtual simulation of London used by Spiers and Maguire (2006). Patient TT showed a mixture of impaired and unimpaired navigation. Some routes were navigated well, others very inaccurately. A variety of 29 different factors were quantified to determine whether any of them explained the pattern of impairments. One of these salient factors was the need to take detours in the environment. Routes that required detours were not more impaired than routes that did, rather it was the requirement to navigate along the minor roads of London that was the most significantly powerful predictor of performance. Thus, the human hippocampus does not appear to be specifically required to take detours in environments well learned prior to the lesion, but when the route requires a less often traveled path it may be needed to aid navigation.

### Hippocampal Contributions based on Single Unit Recording and Lesion Studies in Rodents

**Figure 2:**
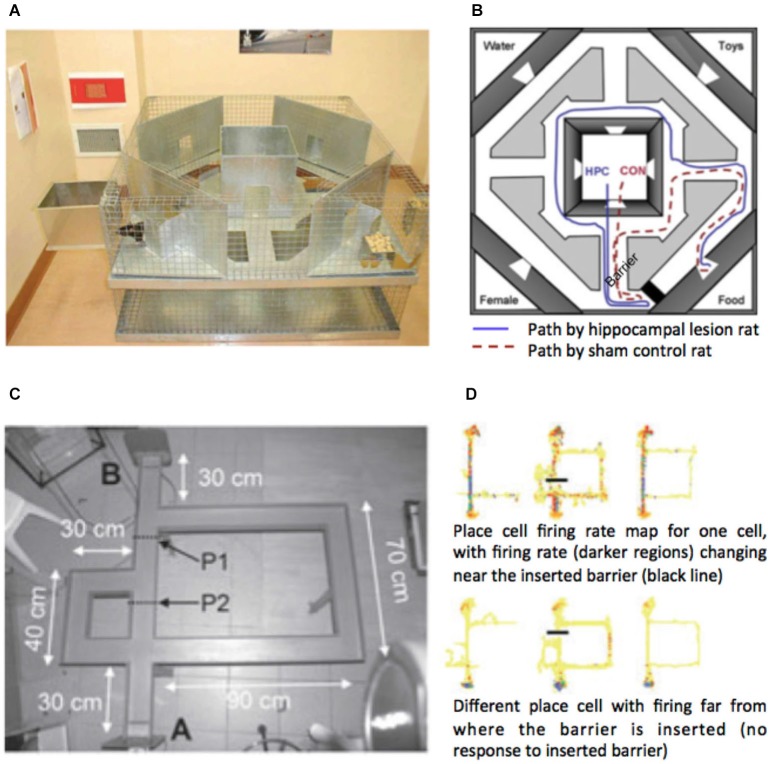
**Detour studies with rodents. (A)** Maze used by Winocur et al. (2010). **(B)** Map of the maze with the path taken by hippocampal lesioned rat and a sham control rat. Black rectangle marks the barrier. Note the longer path taken by the hippocampal lesioned animal. **(C)** Maze used by Alvernhe et al. (2011) adapted from Tolman and Honzik (1930). P1 and P2 mark the points where barriers were inserted on different trials. **(D)** Place cell firing rate maps from two example cells in the study shown before (left), during (middle) and after (right) a barrier has been inserted. Images adapted with permission.

One might imagine, given the extensive research on spatial navigation in rodents, that we would know more about detour processing in the hippocampus from single unit recording or lesion studies. We do not. Much of the large body of research on navigation has explored maze learning, object location learning, or goal switching (e.g., Morris et al., 1982; Packard and McGaugh, 1996; Gilbert and Kesner, 2004; Tse et al., 2007). Only one study, to our knowledge, has examined the impact of hippocampal lesions on a detour task (Winocur et al., 2010), see Figure 2A. The lesioned rats were impaired at making use of optimal detours to reach the goal when a barrier was placed in environment learned 2 weeks prior to lesions. While the control rats were able to readily switch to the next most optimal route, hippocampal rats were not (Figure 2B). They often made errors that persisted over many days. However, their routes were not random. Winocur et al. (2010) noted that the rats made clear purposeful movement to reach the goal and showed “no outward signs of agitation” (p.12). Furthermore, while the rats failed to adopt optimal detours, they were markedly better than a group of rats with hippocampal lesions first exposed to the environment after their lesions. Thus, while non-hippocampal circuits are sufficient for navigation of blocked detours in a variety of situations, damage to the hippocampus can disrupt optimal path taking when explicitly tested. This result is consistent with the recent evidence from patient HC (see Rosenbaum et al., 2015). Current theories would suggest the hippocampus is needed in order to construct detailed precise spatial representations, and the coarse schematic representations of the environment can be formed elsewhere (Moscovitch et al., 2005; Tse et al., 2007; Maguire and Mullally, 2013). Further research using a detour task that requires both fine-grained and coarse-schematic route choices may prove useful in developing a better characterization to the hippocampal contribution.

Similar to lesion studies, single unit recording studies have rarely examined responses to obstructing the path to the goal. Alvernhe et al. examined the impact of forced detours (Alvernhe et al., 2011) and shortcuts (Alvernhe et al., 2008) on the place cell activity in CA1 and CA3 of the dorsal hippocampus. Hippocampal place cells fire action potentials when the animal occupies a specific region of space. The region of space is specific for each cell (referred to as the cell’s place field), allowing the place cells to provide a population code for the whole environment. Evidence indicates that they provide a long-term memory of the environment (Lever et al., 2002). To explore shortcut taking Alvernhe et al. (2008) recorded as rats navigated an M-shaped track for reward in a learned configuration. The shortcut was introduced by having part of the maze removed. To explore detours the researchers tested rats with the paradigm of Tolman and Honzik (1930), in which they initially explore a maze with three possible paths to the goal (Figure 2C). After learning to take the shortest path, this path is blocked and the rats must switch to taking an optimal alternative path. Rats rapidly switch to choosing the second most optimal path, demonstrating some latent learning. In both experiments place cells in areas CA1 and CA3 showed some local remapping of their place fields near the region of the maze with the changed geometry, while cells far from the goal showed little change in their firing rate (Figure 2D). This is consistent with many other studies showing similar place cell responses to geometric change (O’Keefe and Burgess, 1996; Lever et al., 2002; Wills et al., 2005; Spiers et al., 2015), and thus is not a response specific to detour or shortcut taking. However, in the shortcut experiment CA3 cells were found to also show non-local remapping—altering their firing patterns at locations in the maze not near the change. Purely local geometric response models (Hartley et al., 2000) cannot easily account for this change. Instead it may relate to the prospective activity place cells, which change their firing rate depending on the future path of the rat (Wood et al., 2000).

While the studies by Alvernhe et al. (2008, 2011) show how spatial maps in the hippocampus alter in response to changes in the environment that affect the path to the goal it is difficult to relate these findings to neuroimaging data (Table 1). This is because place cells are typically not examined in terms of evoked responses to events. Thus, it is not clear from the data in Alvernhe et al. (2011) how the hippocampal cells responded to the initial discovery of the barrier, and at what point the rat made a decision to change its path. This is also the case for a study by Muir and Taube (2004) in which postsubiculum (dorsal presubiculum) head-direction cells were recorded during the blocked path “sunburst maze” task of Tolman (1948). In this task the rats learn to take a particular set path to a goal. After initial training the learned path is blocked and 8 novel path options are made available, with one of the new paths leading directly to the goal. Tolman (1948) found rats were more likely to take the novel paths oriented to the goal than paths not oriented toward the goal. Because each head-direction cell fires when the rat’s head is oriented in a preferred direction in the environment it is possible to use the variation in their firing as a proxy for how oriented the rats were. Muir and Taube (2004) found no relationship between the consistency of rat’s head-direction representations and their ability to choose optimal novel paths.

Recent developments in large-scale recording have made it possible to decode information about the rat’s position during brief spiking events in open field environments (Pfeiffer and Foster, 2013). These spiking events can show decoding of the rats’ representation of location sweep ahead towards goal locations (Pfeiffer and Foster, 2013). This result is broadly consistent with a mechanism for future path searches using a model-based system (van der Meer et al., 2012). It will be important in future work to explore such activity during forced detour tasks to test whether such spiking event activity is related to future path selection.

### fMRI Evidence for the Contribution of the Hippocampus to Processing the New Path to the Goal

**Figure 3:**
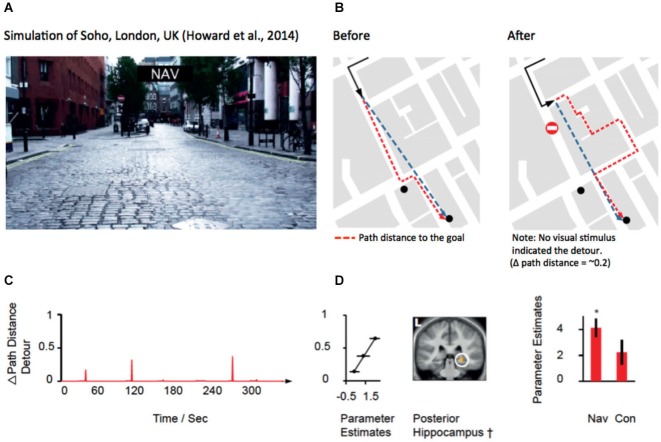
**Hippocampal activity correlated with the change in distance to the goal at a detour. (A)** View from the film simulation of London used by Howard et al. (2014). **(B)** Map of the part of the region navigated. Black line shows the path taken. Red dotted line indicates the optimal future path to be taken to the goal (black dot). Blue dotted line indicates the Euclidean distance to the goal. Left map show the path to the goal before the subject discovers that the simulation has led the subject left rather than allowing them to travel straight. No detour was marked in the film simulation; rather the simulation simply did not take the path the subject had requested prior to the junction. **(C)** The amount of change in the path distance is plotted against time for one of the 10 routes navigated in the experiment. **(D)** Middle: Right posterior hippocampal activity significantly correlated (*p* < 0.05 family-wise error corrected) with the change in path distance at detours is plotted on a mean structural scan, thresholded at *p* < 0.005 uncorrected. Left: Parameter estimates from the peak voxel are plotted for three different ranges of the change in the path distance. Right: parameter estimates of the significant correlation in the navigation condition, but non-significant parameter estimates in the control routes. Note: a significantly greater response was observed in the posterior right hippocampus for navigation routes compared with control routes (see Howard et al., 2014). Images adapted with permission from Howard et al. (2014).

Recent evidence from several fMRI studies has provided convergent evidence that the hippocampus represents the distance to the goal during navigation (Viard et al., 2011; Sherrill et al., 2013; Howard et al., 2014; for review see Spiers and Barry, 2015). Such activation patterns may relate to the forward sweeps of activity observed during travel periods (Johnson et al., 2007; Wikenheiser and Redish, 2015) or brief ensemble spiking events (Pfeiffer and Foster, 2013). More explicitly, Wikenheiser and Redish (2015) have shown that the activity prior to running to a goal is related to the distance to that goal. In the case of detours, the path distance always increases to the goal. For some detours this might be a small change in the distance for others a very large change. To understand how brain regions respond to the change in distance to the goal at detours, Howard et al. (2014) examined activity related to the change in the path distance to the goal (see Figures 3A,B). Figure 3B shows an example of a change in the path needed to reach a goal for one of the detours in the experiment. By plotting the change in the path distance for each second of a route it is possible to identify when various detours occurred and observe how they changed the distance to the goal. Figure 2C shows an example of one of the 10 routes subjects navigated. Right posterior hippocampal activity was found to positively correlate with the amount of change in the detour during navigation routes (Figure 2D). This correlation was absent and significantly lower in the control routes (where subjects did not need to rely on memory to navigate), indicating that the response required goal-directed navigation to be elicited. Notably, no PFC region was correlated with the change in the path distance to the goal. This suggests a division between the PFC for detecting and manipulating information, and the hippocampus for representing information about the path required to reach the goal.

When reconsidering the route to the goal it is possible that the direction to the goal might be computed. Recent evidence suggests that the entorhinal/subicular region represents the allocentric direction to the goal during path planning (Chadwick et al., 2015). Whether this region also represents the direction to the goal when detours are required is unknown. Models of vector navigation argue that such allocentric direction information would be important for guiding the navigator along the optimal new path (Kubie and Fenton, 2009, 2012).

## Conclusion and a Speculative Conceptual Model

**Figure 4:**
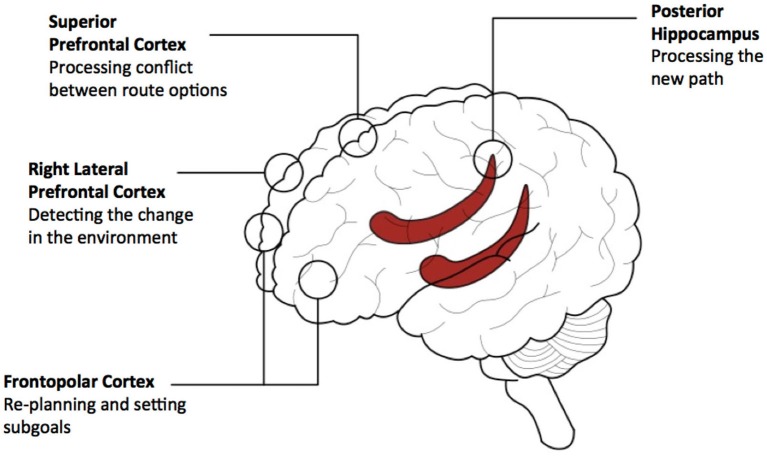
**A conceptual model of prefrontal and hippocampal contributions to navigating detours**. The model provides a summary of the contributions of three prefrontal cortical (PFC) regions and the hippocampus based on our review of empirical data. This model relates to navigating recently learned environments and involves the use of path-based representations for planning. Activity associated with detecting the detour in lateral PFC propagates to the hippocampal circuitry and the other PFC regions to support path-based planning. Activity arising from path processing in the hippocampus propagates to rostral PFC and superior PFC regions for the setting of subgoals and dealing with conflict between possible routes, respectively. Based on computational theories of PFC—hippocampal networks (e.g., Martinet et al., 2011; Hirel et al., 2013), it is predicted that activity would re-route multiple times between anterior/superior PFC regions and the hippocampal network to provide processing of alternative routes, depending on the complexity of the potential route and its options.

Reviewing the limited literature we are able to provide a speculative conceptual model involving: lateral, superior and frontopolar PFC and posterior hippocampus. This model is currently specific to environments that have been learned recently. Lateral PFC is involved in detecting that an alteration in the environment will require a change in the route plan. Frontopolar PFC subsequently provides a mechanism for re-planning, possibly involving the generation of subgoals. The posterior hippocampus pre-activates representations of the future path to the goal to adjudicate between possible routes and superior PFC regions deals with the conflict between options. See Figure 4 for diagram outlining the model.

Replication of the data that supports this division of functions between regions will be invaluable, as will new tests of the model. The current model predicts that the conflict between paths will lead to increased activity in superior PFC, but not other regions. However, it may be that rostral PFC regions also contribute to this function. If frontopolar PFC is related to constructing the new route plan, then the number of subgoals to be considered may correlate specifically with responses in this region. If the hippocampus simulates the future path to the goal then the length of a new shortcut will be expected correlate with hippocampal, but not PFC activity. In addition to these manipulations, future studies teasing apart the involvement of model-based and model-free responses and investigating environments learned in the remote past will be highly beneficial.

A crucial question is how does the PFC interact with the hippocampus during detecting the detour and planning the new route. In our current model we speculate that there is a reciprocal interaction between the hippocampus and the PFC to support this function (Simons and Spiers, 2003; Martinet et al., 2011; Hirel et al., 2013; Preston and Eichenbaum, 2013). Initially, lateral PFC activity propagates to the hippocampal circuitry, and other PFC regions to support path-based planning. Activity arising from path processing in the hippocampus is propagated to rostral PFC and superior PFC regions for the setting of subgoals and dealing with conflict between possible routes. PFC processing may give rise to further perturbation of the hippocampal network to provide more processing of potential routes, which would lead to further processing in PFC and ultimately action. The extent of the interaction between PFC and hippocampus may likely depend on the complexity of the new route to be considered and how much time the individual is willing to invest in considering the options. Formulation of the computations that may be performed in interactions between PFC and hippocampus can be found in the computational models of Martinet et al. (2011) and Hirel et al. (2013). However, currently these models focus on rodent navigation and implement a single PFC region responsible for goal directed route planning. This review points at the need to consider different regions engaged and different situations in which detours may be required (e.g., when expected or not).

## Conflict of Interest Statement

The authors declare that the research was conducted in the absence of any commercial or financial relationships that could be construed as a potential conflict of interest.
